# Seroprevalence and Co-Circulation of Rift Valley Fever Virus and West Nile Fever Virus in Livestock Population of Afar Region, Northeast Ethiopia

**DOI:** 10.1155/2024/8249077

**Published:** 2024-08-26

**Authors:** Jemberu Alemu Megenas, Mengistu Legesse Dadi, Tesfu Kassa Mekonnen, James W. Larrick, Gezahegne Mamo Kassa

**Affiliations:** ^1^ Department of Veterinary Microbiology Immunology and Public Health College of Veterinary Medicine Addis Ababa University, Bishoftu, Ethiopia; ^2^ College of Agriculture and Natural Resources Gambella University, Gambella, Ethiopia; ^3^ Aklilu Lemma Institute of Pathobiology Addis Ababa University, Addis Ababa, Ethiopia; ^4^ Panorama Research Institute, Sunnyvale, CA, USA

## Abstract

The distribution, epidemiology, and socioeconomic impact of Rift Valley fever (RVF) and West Nile (WN) viruses are poorly known in areas of sub-Saharan countries like Ethiopian pastoral region. The human and livestock density in the area has increased greatly in recent years, but little work has been done on arboviral diseases and their potential impact on human and livestock health. The aim of this study was to detect the circulation of zoonotic arboviruses such as Rift Valley fever virus and West Nile viruses in the livestock population and to estimate seroprevalence in Afar pastoral area northeast Ethiopia. Cross-sectional serological survey was carried out in 736 serum samples from which cattle (224), camel (155), goats (121), sheep (144), and donkeys (92) were tested for the presence of anti-RVFV and anti-WNV IgG using a competitive enzyme-linked immunosorbent assay (c-ELISA) in two purposively selected districts of the Afar region. The present study revealed a 9.1% (95% CI = 8.86–9.29) seroprevalence of co-circulation of RVF and WNV. High 32/155 (20.7%) seroprevalence of co-circulation was seen in camels, followed by goat 14/121 (11.6%), cattle 16/224 (7.14%), and sheep 5/144 (3.5%), respectively, and higher 41/421 (9.7%) seroprevalence of co-circulation was observed in *Amibara* district than *Haruka* district. Camels were seven times (OR: 7.016, 95% CI = 2.639–18.653) more likely to be seropositive for the co-circulation than sheep (*p* ≤ 0.001). Livestock herds found in *Amibara* district were 1.2 times (OR: 1.165, 95% CI = 0.680–1.996) more likely to be seropositive for RVFV infection than those in *Haruka* areas. Given the co-occurrence of RVFV and WNV circulations, along with often suboptimal human and animal health surveillance in many similar areas' attention should be given. Investigation of the potential socioeconomic and health impacts of zoonotic arbovirus infections in such areas is crucial. Since both RVFV and WNFV are transmitted through a mosquito vector, avoiding mosquito bites is the primary method of prevention.

## 1. Introduction

Members of the families Flaviviridae, Togaviridae, Phenuviridae, Peribunyaviridae, Reoviridae, Asfarviridae, Rhabdoviridae, Orthomyxoviridae, and Poxviridae are among the diverse group of vector-borne pathogens known as arboviruses [1]. The most significant viruses that infect vertebrates are those belonging to the Bunyaviridae family which causes Rift Valley fever and Flaviviridae families which causes West Nile fever. These diseases are emerging or re-emerging in the 21st century and are extremely important for veterinary and public health [2, 3].

Rift Valley fever is a vector-borne zoonotic disease caused by the Rift Valley fever virus (*Phlebovirus* genus), listed among the eight pathogens included in the Bluepoint list by the WHO [4]. The transmission is primarily carried out by Aedes and Culex mosquito species [5, 6]. Symptoms of the disease are varied and nonspecific, making clinical diagnosis often challenging, especially in the early stages [7]. Early diagnosis of Rift Valley fever is crucial to limit its spread and provide appropriate care to patients, as it is difficult to distinguish from other viral hemorrhagic fevers and many other febrile diseases [4, 8, 9].

Many African nations as well as the Arabian Peninsula are endemic to this disease [10–12]. Although it is currently limited in its geographic range, the Rift Valley fever virus has the potential to spread to previously unaffected regions, such as Ethiopia. This illness is one of the major viral zoonoses in Africa; epizootics are typified by fetal deformities, abortion storms, and newborn animal deaths in ruminant livestock. Significant outbreaks result in significant financial losses because of livestock mortality and trade restrictions [12].

Although West Nile virus (WNV) is also an important zoonosis and transmitted by arthropods, the virus is caused by a flavivirus in the family Flaviviridae [13–15]. It was first isolated in 1937 from the blood of a woman in the West Nile province of Uganda who had a mild febrile illness [16]. Since then, it has been associated with sporadic and major outbreaks in humans and horses, as frequent outbreaks with an increased proportion of neurological disease cases have been reported [14, 17–19]. The virus is maintained in an enzootic cycle between ornithophilic mosquitoes and birds by infected Culex spp. mosquitoes [9].

Both Rift Valley fever and West Nile viruses are primarily transmitted by Aedes and culex mosquitoes, respectively, and several bioclimatic environments such as dambos, semiarid, and irrigation regions are suitable for RVF and WNV emergence. The distribution, epidemiology, and socioeconomic impact of Rift Valley fever (RVF) and West Nile (WN) viruses are poorly known in sub-Saharan African countries like Ethiopian pastoral areas. The human and livestock density in the area has increased greatly in recent years, but little recent work has been done on arboviral diseases and their potential impact on human and livestock health. The aim of this study was to detect co-circulation of RVF and WNV zoonotic arboviruses in the livestock population and estimate seroprevalence in Afar pastoral area of northeast Ethiopia.

## 2. Materials and Methods

### 2.1. Description of the Study Area and Study Design

The study was conducted in the Afar pastoral region, northeastern Ethiopia from June 2021 to April 2022. Samples were collected in the districts of Amibara and Haruka, which are close to the Awash River basin and situated along migratory bird migration routes, both of which are significant factors in the virus's epidemiology. The study districts were chosen due to their large livestock populations, water bodies, irrigation activities, frequent flooding, evidence of mass abortion, and retained placenta. A cross-sectional study design was employed to evaluate the seroepidemiological rate of the co-circulation of RVFV and WNV infections.

### 2.2. Study Animals

The study included cattle, camels, goats, sheep, and donkeys in the two districts of the Afar pastoral region.

### 2.3. Sample Size Determination and Sampling Method

The desired sample size was calculated according to Thrusfield (2005) sample size determination formula as follows:

(1) $$\begin{array}{c} n=\frac{1.96^{2}\text{xpexp}\left(1-\text{pexp}\right)}{d^{2}}, \end{array}$$

where *n* = required sample size, Pexp = expected prevalence = 50%, and *d* = desired absolute precision = 0.05.

As a result of having no previous study of the disease prevalence rate in the region 50% expected prevalence was employed, and the sample size was 384 for each of the study districts and a total of 736 samples were collected. Data such as age, sex, history of mass abortion, history of mass death of young animals, and selected clinical signs for each animal were recorded separately using a provisional paper-based identification code. This information was documented during blood collection using a checklist prepared as a mini questionnaire.

### 2.4. Sampling Procedure and Data Collection

Upon obtaining informed consent from local elders and animal owners, a haphazard sampling technique was employed for selecting animals within the herds, given the absence of livestock registration in the study area. In the selected households, individuals aged over 18, and who provided informed consent, were interviewed.

### 2.5. Blood Sample Collection

A sterile plain vacutainer tube with a needle was used to puncture the jugular vein of selected animals in order to obtain a 5 ml blood sample, following informed consent from local elders and animal owners and following OIE guidelines. Until a clot formed, the drawn blood samples were labeled and stored at room temperature. The serum samples were kept at −20°C after being centrifuged for 10 minutes at 3000 rpm.

### 2.6. Serological Analysis

The detection of anti-RVFV and anti-WNFV-IgG antibodies was performed using the ID screen® RVFV and WNV competition multispecies ELISA kits (ID-Vet Innovative Diagnostics Montpellier, France). Results were assessed at an optical density (OD) of 450 nm using a 96-well ELISA plate reader (Multiskan™ FC Microplate Photometer). The results were interpreted as either positive or negative based on the manufacturer's recommended cut-off values.

### 2.7. Ethical Considerations

The research project underwent thorough review by the Animal Research Ethics Committee of the College of Veterinary Medicine and Agriculture at Addis Ababa University referenced (VM/ERC/40/03/15/2023). Informed consent was obtained from the animal owners, and adherence to the ARRIVE criteria [20] was maintained throughout the entire study process.

### 2.8. Data Analysis

Descriptive and inferential analyses were conducted using *R* software version 4.0.3 for Windows via RStudio Version 1.3.1093 and Microsoft Office™ Excel® 2019 spread sheets. Mean differences of variables were tested using one-way ANOVA and Student's *t*-test. Bivariate analysis utilized the Pearson Chi-Square test of association and Fisher's exact test when appropriate, with a significance level set at *p* ≤ 0.05. Univariable linear regression models were generated to assess multicollinearity for the expected outcome of the ELISA test results. Subsequently, a multivariable linear regression model was constructed, incorporating variables that retained significance (*p*  < 0.05) in the univariable linear regression analysis. The resulting multivariable model underwent testing for goodness of fit and predictability through the Hosmer–Lemeshow test and Omnibus test, respectively. All potential risk factors in the model exhibited *p* values less than 0.05, signifying their statistical significance in the analysis.

## 3. Results

The study focused on detecting anti-IgG Rift Valley and anti-IgG West Nile antibodies in a total of 736 serum samples of which 155 were from camels, 224 from cattle, 92 from donkeys, 121 from goats, and 144 from sheep.

### 3.1. Seroprevalence of Rift Valley Fever and West Nile Fever Viruses Co-Circulations

The present study revealed a 9.1%, 95% CI = (8.86–9.29) seroprevalence of co-circulation of RVF and WNV in the study area at the same time (Table 1).

### 3.2. Factors Associated with Co-Circulation of RVF and WNV

There was a statistical difference in species and location for the seroprevalence of co-circulation. Camels showed a higher seroprevalence of 20.7% (32/155) compared to other animal species in the study area (*p* ≤ 0.001). Additionally, the Amibara district exhibited a higher seroprevalence of 9.7% (41/421) (Table 2).

Camels were seven times (OR: 7.016, 95% CI = 2.639–18.653) more likely to be seropositive for the co-circulation than sheep (*p* ≤ 0.001). Livestock herds found in *Amibara district* were (OR: 1.165, 95% CI = 0.680–1.996) 1.2 times more likely to be seropositive for RVFV infection than those in *Haruka* areas (*p* ≤ 0.001) (Tables 3 and 4).

## 4. Discussion

The present study reports a notably high seroprevalence of IgG to RVFV and WNV co-circulation in the livestock population, with 9.1% of the sampled livestock testing positive. This prevalence is significantly higher compared to similar studies conducted in Brazil [21]. Co-circulation of multiple viruses could occur through either simultaneous transmission during a single mosquito bite or through successive mosquito bites. Epidemiological synergy between outbreaks of viruses transmitted by mosquitoes, such as chikungunya, dengue, and Zika viruses, has resulted in coinfection of humans with multiple viruses [22]. Previous study showed evidence of co-circulation of multiple arboviruses transmitted by *Aedes* species based on laboratory syndromic surveillance at a health unit in a slum of the Federal District, Brazil [21].

Arboviruses, like the Rift Valley fever virus (RVFV) and the West Nile virus (WNV), have emerged and reemerged in formerly uninhabitable regions of the globe [23]. In sub-Saharan Africa, particularly in Ethiopia, a hotspot for arbovirus occurrence, there has been a significant increase in the density of people and livestock in the area recently, little research has been done on arboviral diseases in Ethiopia. Coinfections in Pastoral areas are likely to rise with the burden of arbovirus. The intricacies of these coinfections and their effects on the health of humans and animals are not well understood [24–26]. When different viruses are transmitted by one or more vectors at the same time, the resulting infections may have unpredictable effects [21]. Dynamics of disease and patterns of transmission may be greatly impacted by such complexities. Thus, the purpose of this study was to determine the seroprevalence of RVFV and WNV in livestock population in the Afar pastoral area as well as to investigate the presence of co-circulation of these two viruses.

Rift Valley fever and West Nile virus infections are classically considered “endemic” in Africa, especially sub-Saharan Africa, but the precise situation of the disease in Ethiopia, has not been established well. The present study results obtained made it possible to highlight the presence of IgG–Rift Valley fever and West Nile in livestock sera in the districts of Amibara and Haruka, Afar pastoral area. Indeed, very few studies [27, 28] have taken into account the presence of the virus in Cattle population of Gambella and South Omo Ethiopia, respectively.

The current investigation demonstrated that there was a seroprevalence of 0% for Rift Valley fever virus (RVFV) in donkeys, indicating that donkeys in the studied region do not carry RVFV. This outcome corresponds with research from Egypt as documented by [9, 29], where the overall seroprevalence of co-circulation was also found to be 0%. The seroprevalence findings suggest that domestic livestock serve as valuable sentinels for both RVF [30] and WNV [19], although the underlying biological mechanism for this phenomenon remains unclear. This conclusion is supported by the work of Selim, Abdelhady [31]. Additionally, Cichon, Barry [32] previously described a co-infection of the ruminant population with RVFV and NRIV in Kenya and Somalia during 1997–1998.

There is statistical difference in species and location for the seroprevalence of the co-circulation and higher 32/155 (20.7%) seroprevalence of co-circulation was exhibited in camels compared with other species of animals in the study area, *p* ≤ 0.001. Higher 41/421 (9.7%) seroprevalence of co-circulation was observed in Amibara district. The reason for this relatively high seroprevalence among animals in the study area could be due to the biological nature of camel and goats are browsers that mosquitoes used for resting during higher temperature and could be it is a mosquitoes breeding site, samples were taken in the near proximity of Awash River basin causes frequent flooding and its geographical location on the routes of migratory birds that play an important role in the epidemiology of the virus. These findings may be attributed to the domestic animals present in large herds which attract a greater number of mosquitoes which is supported by [33]. It is interesting to note that RVF and WNV antibodies were detected in most of the domestic animals except in donkeys in the study districts playing a role in the maintenance and circulation of the virus among animals.

Multiple factors impact the transmission and distribution of WNV. Among drivers, weather conditions have direct and indirect influences on vector competence, on the vector population dynamic and on the virus replication rate within the mosquito. Temperature plays an important role in viral replication rates and transmission of RVF and WNV [3].

In this study, the determining factors that were found to be significantly associated with RVF and WNV exposure in the logistic regression analysis were the species difference and location of the study. The probability of co-circulation in camels was seven times higher than that in sheep (OR = 7.302, 95% CI 2.757–19.339, *p* ≤ 0.001), and the likelihood of co-circulation in goats was 3.75 times greater than in sheep (OR = 3.746, 95% CI 1.304–10.763, *p* ≤ 0.001). This variance could be attributed to the browsing behavior of camels and goats, potentially providing resting habitats for mosquitoes in the study region. The probability of co-circulation in the Amibara district (OR = 1.202, 95% CI: 0.706–2.045) was 1.2 times greater than that in livestock located in the Haruka district, with a significant *p* value of *p* ≤ 0.001. This discrepancy could potentially be attributed to several factors present in the Amibara district, including the existence of a wildlife sanctuary and a national park, as well as larger livestock herds and the mingling of various livestock species. These conditions may create an environment conducive to the transmission of arboviruses via mosquito bites.

## 5. Conclusion

The present study reports a notably high seroprevalence of IgG to RVFV and WNV co-circulation in the livestock population, with 9.1% of the sampled livestock testing positive. Given the ongoing changes in environmental and societal factors, the threat of arboviral outbreaks such as RVFV and WNFV remains significant. Collaborative efforts among governments, healthcare entities, and researchers are essential to develop effective surveillance, prevention, and response measures. Conducting thorough research using a confirmatory test like rPCR, is advised to validate the presence of circulating infectious agents.

## Figures and Tables

**Table 1:** Seroprevalence of RVFV and WNV co-circulation in livestock populations.

Species	No. of animals sampled	No. positive (%)	95% CI	*p* value
Camel	155	32 (20.7)	18.01–22.03	*p* ≤ 0.001
Cattle	224	16 (7.14)	5.91–9.23	*p* ≤ 0.001
Donkey	92	0 (%)		*p* ≤ 0.001
Goat	121	14 (11.6)	9.09–12.99	*p* ≤ 0.001
Sheep	144	5 (3.5)	1.92–4.13	*p* ≤ 0.001
Overall	736	67 (9.1%)	8.86–9.29	*p* ≤ 0.001

*p* ≤ 0.001 statistically significant.

**Table 2:** Factors associated with seroprevalence of antibodies against RVF and WNV for livestock populations of Afar pastoral area, 2022.

Variable	Category	No. of animals sampled	No. positive (%)	95% CI	Chi-square	*p* value
Species	Camel	155	32 (20.7)	18.01–22.03		
	Cattle	224	16 (7.14)	5.91–9.23		
	Donkey	92	0		41.616	*p* ≤ 0.001
	Goat	121	14 (11.6)	9.09–12.99		
	Sheep	144	5 (3.5)	1.92–4.13		

Sex	Male	155	13 (8.4)	5.8–10.9	0.122	0.725
	Female	581	54 (9.3)	6.03–12.01		
	<2 years	74	8 (10.8)	7.1–11.9		

Age	2–<5 years	208	24 (11.5)	9.02–13.09	3.60	0.308
	5–<10 years	332	28 (8.4)	6.03–10.04		
	>10 years	122	8 (6.6)	4.32–8.05		

Location	Amibara	421	41 (9.7)	6.12–13.02	739.12	*p* ≤ 0.001
	Haruka	315	26 (8.3)	7.02–10.01		

**Table 3:** Binary logistic regression analysis of potential risk factors associated with anti-RVFV and WNV IgG seropositivity among livestock in the Afar pastoral area of Ethiopia 2022.

Variable	Category	No. of animals sampled	No. positive (%)	Sig	COR	95% CI	
						Lower	Upper
Species	Camel	155	32 (20.7)	*p* ≤ 0.001	7.016	2.639	18.653
	Cattle	224	16 (7.14)	*p* ≤ 0.001	2.056	0.732	5.773
	Donkey	92	0				
	Goat	121	14 (11.6)	0.997	3.631	1.256	10.497
	Sheep	144	5 (3.5)	0.017	Ref	Ref	Ref

Sex	Female	581	54 (9.3)	0.305	0.981	0.507	1.896
	Male	155	13 (8.4)	0.157	Ref	Ref	Ref

Age	<2 years	74	8 (10.8)	0.083	2.206	0.737	6.603
	20–<5 years	208	24 (11.5)	0.301	2.207	0.902	5.398
	5–<10 years	332	28 (8.4)	0.954	1.586	0.662	3.800
	>10 years	122	8 (6.6)	0.578	Ref	Ref	Ref

Location	Amibara	421	41 (9.7)	*p* ≤ 0.001	1.165	0.680	1.996
	Haruka	315	26 (8.3)	*p* ≤ 0.001	Ref	Ref	Ref

COR, crude odds ratio; Ref, reference point.

**Table 4:** Multivariate logistic regression analysis of potential risk factors associated with anti-RVFV and WNV IgG seropositivity among livestock in the Afar pastoral area of Ethiopia 2022.

Variable	Category	No. of animals sampled	No. positive (%)	Sig	AOR	95% CI	
						Lower	Upper
Species	Camel	155	32 (20.7)	*p* ≤ 0.001	7.302	2.757	19.339
	Cattle	224	16 (7.14)	*p* ≤ 0.001	2.166	0.775	6.054
	Donkey	92	0				
	Goat	121	14 (11.6)	0.996	3.746	1.304	10.763
	Sheep	144	5 (3.5)	*p* ≤ 0.001	Ref	Ref	Ref

Location	Amibara	421	41 (9.7)	*p* ≤ 0.001	1.202	0.706	2.045
	Haruka	315	26 (8.3)	*p* ≤ 0.001	Ref	Ref	Ref

Ref, reference point.

## Data Availability

The data used to support the findings of this study are currently under embargo while the research findings are commercialized. Requests for data, (6/12 months) after publication of this article, will be considered by the corresponding author.
